# Progress on the national echinococcosis control programme in China: analysis of humans and dogs population intervention during 2004–2014

**DOI:** 10.1186/s40249-020-00747-7

**Published:** 2020-10-02

**Authors:** Qing Yu, Ning Xiao, Shuai Han, Tian Tian, Xiao-Nong Zhou

**Affiliations:** 1 Department of Echinococcosis, National Institute of Parasitic Diseases, Chinese Center for Disease Control and Prevention, Shanghai, 200025 China; 2 Chinese Center for Tropical Diseases Research, Shanghai, 200025 China; 3 WHO Collaborating Centre for Tropical Diseases, Shanghai, 200025 China; 4 National Center for International Research on Tropical Diseases, Ministry of Science and Technology, Shanghai, 200025 China; 5 Key Laboratory of Parasite and Vector Biology, Ministry of Health, Shanghai, 200025 China; 6 National Institute of Parasitic Diseases, Chinese Center for Disease Control and Prevention, Shanghai, 200025 China

**Keywords:** Control progamme, Effectiveness, Echinococcosis, China

## Abstract

**Background:**

A national control program for echinococcosis has been in effect since 2005 in China. This program has applied a comprehensive strategy, and good control results have been achieved. Human echinococcosis prevalence rate decrease from 1.08% in 2004 to 0.24% in 2012. The objective of this study is focusing on assessment of the programme with two indices, including patient treatment and registered dogs deworming, in endemic areas of echincoccosis control over the period of 10 years (2004–2014) in China.

**Methods:**

We established the database including demography at county and township levels with coverage for ten provinces and autonomous regions of China in this study. We using methods of epidemiological descriptive, instead the expectation-maximization for missing value filling for grouping available patients into those subjected to surgery and those receiving drug treatment after population screening and the dogs population after registered by deworming. We performed Microsoft Excel software and SPSS software on the results as percentages with the corresponding 95% confidence intervals (95% *CI*s). We also statistically analyzed the economics data on patient treatment and dogs deworming after the corresponding discount with annual bank interest rates (USD 1 = CNY 6.5, bank discount average changes of 2.3–3.3%).

**Results:**

During 2004–2014, the grant total average rate of surgical patient (after surgical operation) treatment had increased with 32.4% and with 81.3% for medical treatment with albendazole. Meanwhile, it increased by 58.6% for the deworming of registered dog since 2007. The accumulated costs amounted to USD 27.03 million after discount for patients and registered dog treatment, which is 1/4 of the total accumulated financial inputs (USD 110.67 million from the Chinese Government). Since the implementation of the national program, it has increased 57 times with respect to the annual financial inputs (costs) and 368 times with respect to all accumulated financial inputs (costs).

**Conclusions:**

This study showed that in endemic areas, patient diagnosis and management, dog management and treatment over this period helped reduce the parasite load to control the disease. More attention should be paid to controlling wild canines during the ongoing program period and sustainable follow-up evaluations are crucial for success and continued implementation of the national program.

## Background

Echinococcosis (hydatid disease) is one of the 17 neglected tropical diseases recognized by the World Health Organization (WHO) and its continued spread is a severe public health concern [1]. Echinococcosis is mainly endemic in areas of central, eastern and western Asia, South America, Oceania and southern, northern and eastern Africa. Infection in humans/livestock/small mammals is caused by the larval stage of the parasite. Canines (i.e. dogs, foxes, and wolves) are the definitive hosts and play a key role in the transmission and dissemination of the adult stage of this tapeworms belonging to genus *Echinococcus*. The two most important zoonotic species, *E. granulosus* (the causative agent of cystic echinococcosis - CE) and *E. multilocularis* (the causative agent of alveolar echinococcosis - AE), are a serious threat to over 1 million people and responsible for over USD 3 billion in expenses every year. Expressed in global disability-adjusted life years (DALYs), losses of 0.3–1 million DALYs for CE and 0.65 million DALYs for AE, respectively, have been reported [2–5].

China has a high prevalence of human CE and one of the highest prevalence levels of human AE, accounting for 40% of global DALYs lost worldwide [4, 6–8]. Both diseases are widely endemic in the pastoral and farming-pastoral regions of Inner Mongolia, Sichuan, Yunnan, Tibet, Shaanxi, Gansu, Qinghai, Ningxia, and Xinjiang provinces/autonomous regions covering 350 counties, with 120 000 people infected and 50 million people estimated at risk according to the national prevalence survey of echinococcosis initiated by the National Ministry of Health in 2012 [9]. Notably, canine infectious sources in wild environments in the above endemic areas play important roles in the transmission dynamics of both diseases [10–13].

A national program for echinococcosis control with comprehensive approaches was launched in 2005, that led by National Ministry of Health was implemented in sectoral collaboration with 13 other ministries, e.g., the Ministry of Agriculture and the Ministry of Water Resources. The activities consisted of health education, sanitation improving, ultrasound screening of the human population, surgical interventions and treatment with albendazole, including management and deworming of the dog population (8 times above per year). This program has developed well, particularly thanks to the national control action plan (2010–2015) implemented by the Chinese Government, as evidenced by the human echinococcosis prevalence rate, which has shown a remarkable decrease from 1.08% in 2004 to 0.24% in 2012 [9, 14–16]. The key measures were monthly praziquantel oral treatment (reported to have 99.9% efficacy by WHO) of registered dogs in addition to large-scale human surgical interventions and oral treatment in the endemic regions [17]. Clearly, control of CE and AE will have important economic consequences. An evaluation of the outlays of the human intervention and deworming of the dog population is essential and should be part of any program aimed at the control of parasitic zoonoses [18]. The objective of this study was to assess progress and outlays for the national echinococcosis control program at the central government level over a 10-year period up to 2014. This assessment includes a comprehensive, epidemiological description and economics analysis with estimates for human and dog interventions in the endemic areas of China for the period 2004–2014.

## Methods

### Study area

**Fig. 1:**
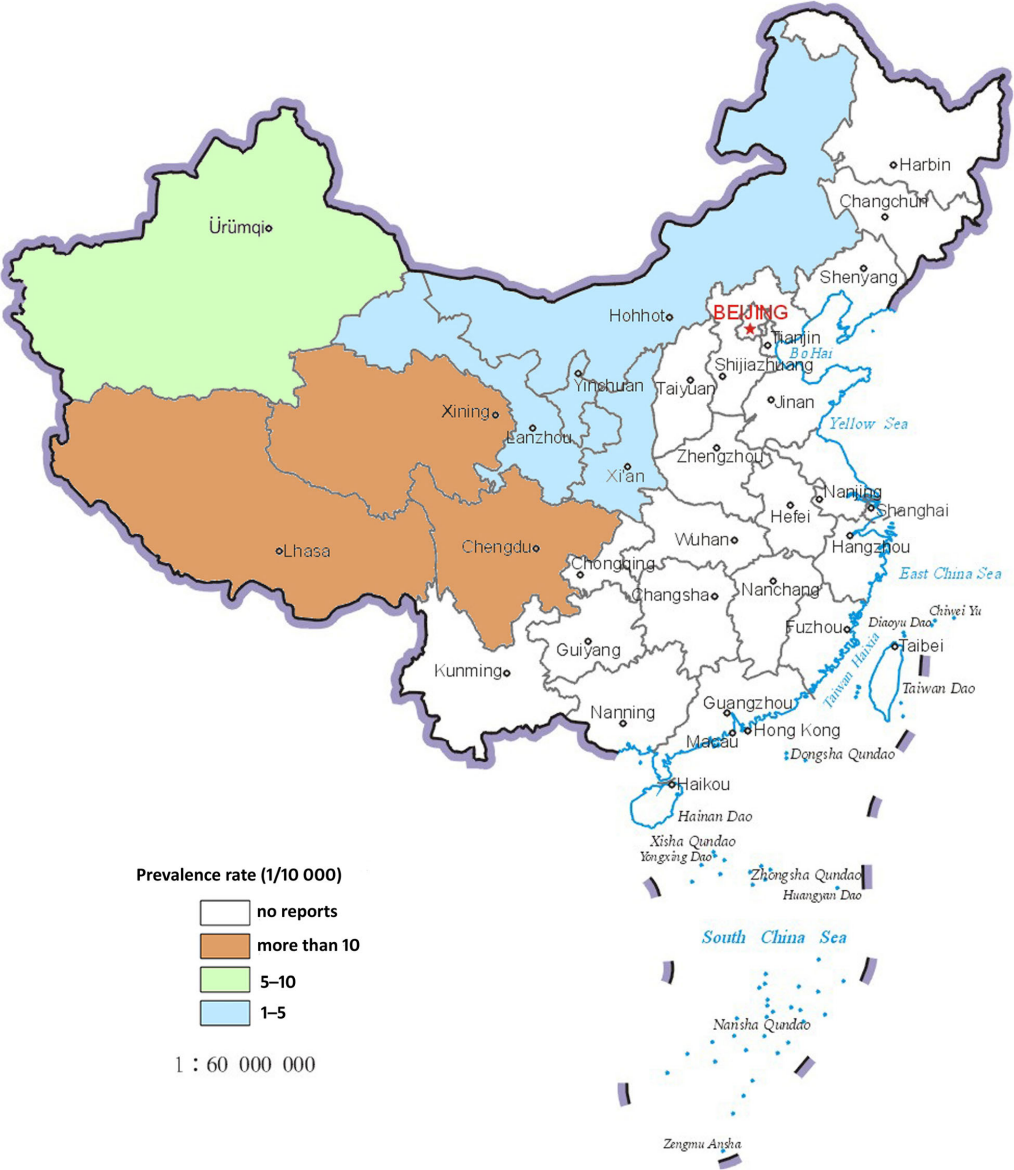
Geographic distribution of human cases of cystic echinococcosis (CE) reported by the Chinese system for infectious diseases in 2014

**Fig. 2:**
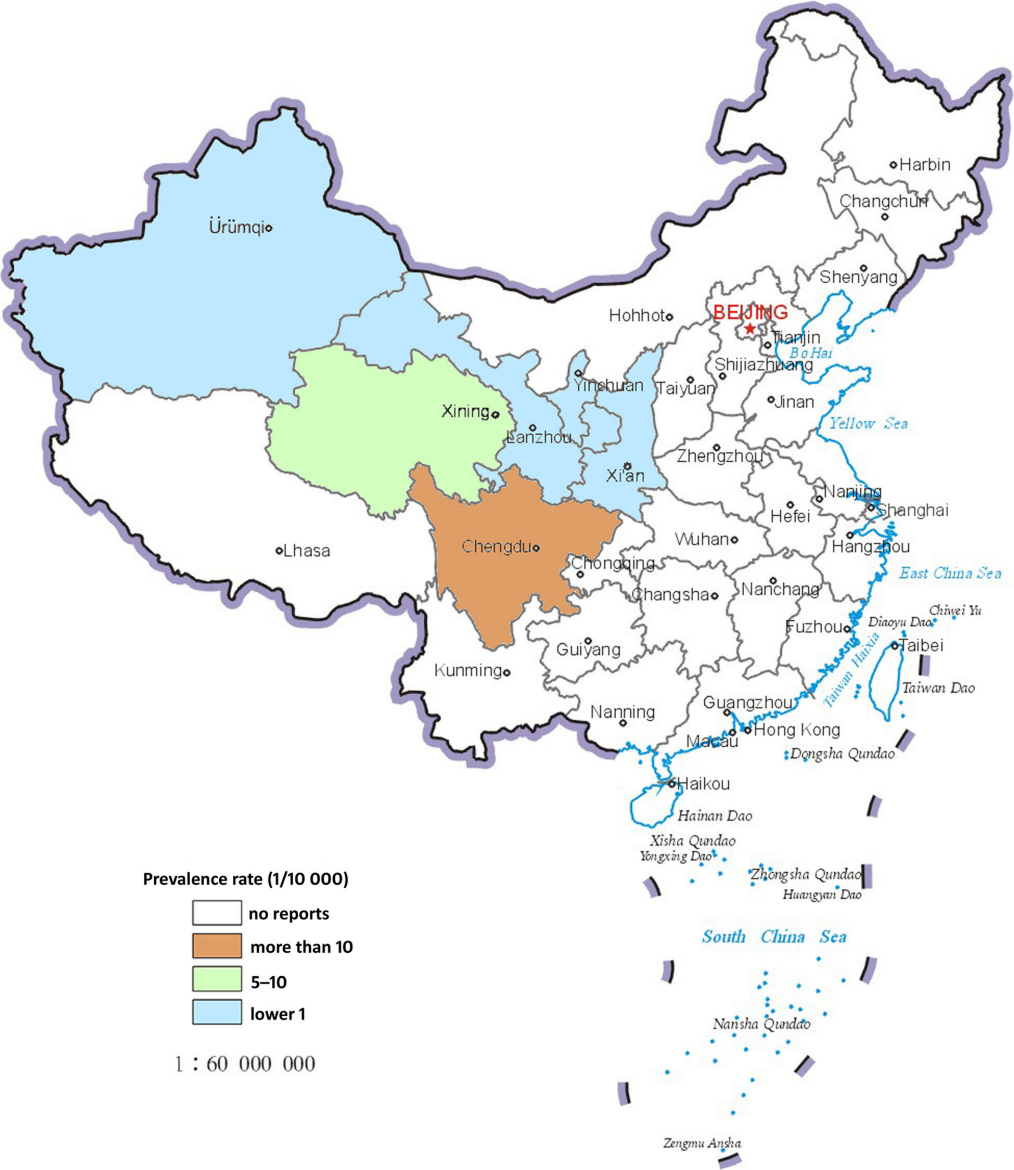
Geographic distribution of human cases of alveolar echinococcosis (AE) reported by the Chinese system for infectious diseases in 2014

Based on the National Control Plan on the Prevention and Control of Key Parasitic Diseases (2006–2015) [15], the study was conducted in the pastoral and farming-pastoral regions of project counties in ten provinces and autonomous regions (Inner Mongolia, Sichuan, Yunnan, Tibet, Shaanxi, Gansu, Qinhai, Ningxia, Xinjiang and the Xinjiang Production and Construction Corps) where the echinococcosis control measures were implemented and supported by budgeted annual financial requirements by the Chinese Central Government covering the period 2004–2014 (Figs. 1 and 2).

### Data collection

All data were obtained in the form of echinococcosis control statistics from the disease-endemic areas approved by the National Ministry of Health for each year of the 10-year period. Data involving patient privacy were not involved. Based on the collected information, a database was established that included demography at county and township levels, grouping available patients into those subjected to surgery and those receiving drug treatment after population screening. Information was given regarding the diagnosis (CE, AE, co-infection or unclassified) and the number of registered dogs and that of those dewormed. The situation with respect to humans and dogs were described using epidemiological descriptive analyses and the direct costs calculated after discount.

### Data analysis

#### Statistical analysis on human and dog intervention

Epidemiological descriptive for human and dogs population were performed using Microsoft Excel software, version 2016 (Microsoft Office, CA, USA) and SPSS software version 22 (SPSS Institute, Chicago, IL, USA). The maps for the distribution of human echinococcosis (CE and AE) prevalence in 2014 were generated using ArcGIS software, version 10 (ESRI, Redlands, CA, USA). The results are shown as percentages with the corresponding 95% confidence intervals (95% *CI*s). The annual numbers of dewormed dogs were not directly acquired for the period 2012–2014 due to a change in the statistical criterion adjustment in these years; instead the expectation-maximization (EM) method for missing value filling (https://en.wikipedia.org/wiki/Expectatio-maximization_algorithm) was used to estimate the number of dewormed dogs statistically with reference to the annual total of the registered dog variable and the human prevalence rate for 2004–2011.

#### Statistical analysis on the outlays

The annual financial outlays for the control activities were allocated at the province and county level by the Chinese Central Government. The methods of economics deviations and proportion comparison were used for the outlays of patients’ treatment and dogs deworming statistics and analyzed. The outlays after discount of each documented surgical and treatment patient was in accordance with the national available direct financial inputs approved by the State Ministry of Finance as follows (using the exchange rate of USD 1 = CNY 6.5): the annual financial inputs calculated with bank discount average changes of 2.3–3.3%: USD 1230 per surgical case; and USD 1.20 per treatment case at the beginning of 2006 [19]. Additionally, six formulas were expressed as follows for the estimated gap analysis between estimated and actual needs on available patients and registered dogs population calculated and analysis.

1. The annual gap for treatment of patients (A1) = the annual number of available patients in need of treatment – the actual annual cost for patient treatment based on cost, actual number of patients and treatment (CAT);
2. The annual gap for dog deworming (A2) = the annual number of registered dogs needing deworming – the cost for annual actual dogs deworming based on cost, actual number of dogs and deworming (CAD);
3. The accumulated gap for patient treatment (A3) = The accumulated number of available patients in need of treatment – the actual accumulated cost for patient treatment based on cost, actual number of accumulated patients and treatment (CAAT);
4. The accumulated gap for dog deworming (A4) = the accumulated number of registered dogs needing deworming) – the actual accumulated cost for dog deworming based on cost, actual number of accumulated dogs and deworming (CADD);
5. Grand annual proportion = A1 + A3/ CAT+CAD; and
6. Grand accumulated proportion = A2 + A4/CAAT+CADD.

### Ethical statement

The database is original from National Institute of Parasitic Diseases, China CDC that authorized by National Ministry of Health, China.

## Results

### Humans intervention

During the period 2004–2014, the total number of human echinococcosis cases reported in the endemic areas ranged from 1749 to 31 507 among 56.7–79.7 million inhabitants at the county level and 19.3–39.1 million inhabitants at the township level. The grand total included individuals identified by annual mass screening (4.8% [959/20 168]–18.2% [318/1749]), the number of patients subjected to surgical intervention (38.1% [938/2461]–91.1% [12 572/24 733]) and patients receiving treatment (53.4% [1313/2461]–98.0% [20 196/20 624]). The statistical analysis of the patients showed that although the average rate of surgical operations for 2005–2006, i.e. before the implementation of the national control program, was greater than 10%, the rate declined to 5.7% in 2007 (95% *CI*: 5.0–6.9%) and remained stable until 2014. The average coverage rate (the grant total average rate) of patients under treatment was 69.4% (95% *CI*: 56.8–82.0%) in 2007, while the total coverage rate was 76.7% (95% *CI*: 67.5–87.9%) in 2014; these rates were stable over the whole study period (Table 1).

**Table 1:** Patient interventions undertaken by the national echinococcosis control program during 2004–2014

Year	Project county^**a**^	Project township^**b**^	Population examined^**c**^	Available patients	Proportion of patients subjected to surgical interventions (%)					Proportion of patients treated with albendazole (%)					Grand total^**d**^ (%)
					CE	AE	Co-infection	Unclassified	Average	CE	AE	Co-infection	Unclassified	Average	
2004	10	70	2.8	1749	17.7	0.5	–	–	18.2	36.5	10.2	–	–	46.7	64.9
2005	25	116	4.9	2461	14.9	0.4	–	–	15.2	27.7	10.4	–	–	38.1	53.4
2006	38	192	12.0	3920	9.5	0.5	–	–	10.0	28.0	15.5	–	–	43.5	53.5
2007	67	370	39.1	7399	5.6	0.1	–	–	5.7	41.2	27.7	–	–	69.4	75.1
2008	103	591	52.8	14 227	4.4	0.2	–	0.2	4.8	39.3	25.8	–	14.4	79.4	84.2
2009	118	899	83.4	18 108	3.1	2.1	–	0.0	5.1	49.8	28.7	–	3.9	82.3	87.4
2010	120	904	90.0	20 168	4.3	0.0	0.4	0.05	4.8	57.2	19.4	6.0	6.4	88.9	93.7
2011	175	1455	152.2	20 624	5.9	0.1	0.0	0.7	6.8	27.8	4.5	0.1	58.9	91.1	97.9
2012	214	2270	166.6	24 733	4.8	0.1	0.02	0.8	5.7	28.8	4.5	0.2	50.8	84.3	90.0
2013	230	2333	183.8	28 652	6.9	0.3	0.02	0.2	7.4	50.9	17.9	0.9	0.5	70.2	77.6
2014	247	2624	213.0	31 507	6.9	0.3	0.1	0.3	7.6	47.9	18.5	0.9	1.9	69.1	76.6

*CE* cystic echinococcosis, *AE* alveolar echinococcosis

^a^ number of counties subjected to control activities

^b^ number of townships subjected to control activities

^c^ **(**×10 000**)**

^d^ proportion of patients treated by surgical interventions+patients treated with albendazole

--means no record

**Fig. 3:**
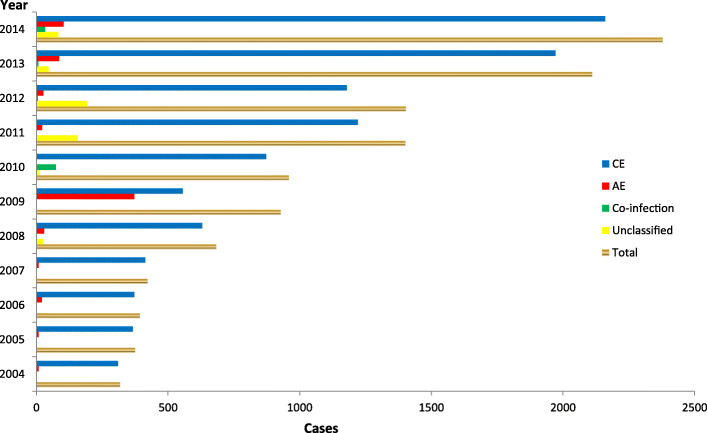
Classification of patients subjected to surgical interventions in the period 2004–2014

**Fig. 4:**
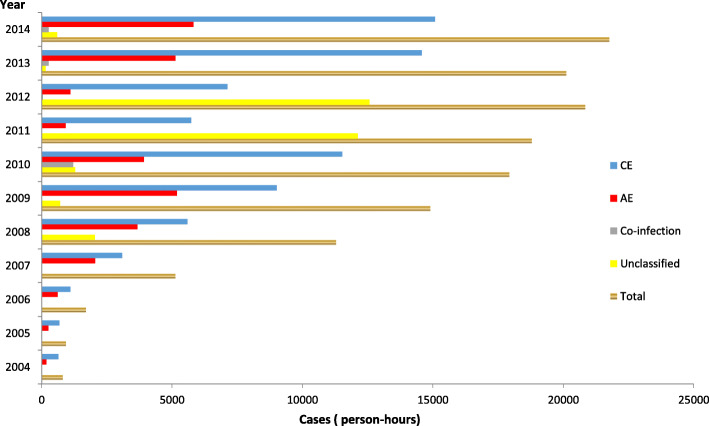
Classification of patients receiving albendazole treatment in the period 2004–2014

**Fig. 5:**
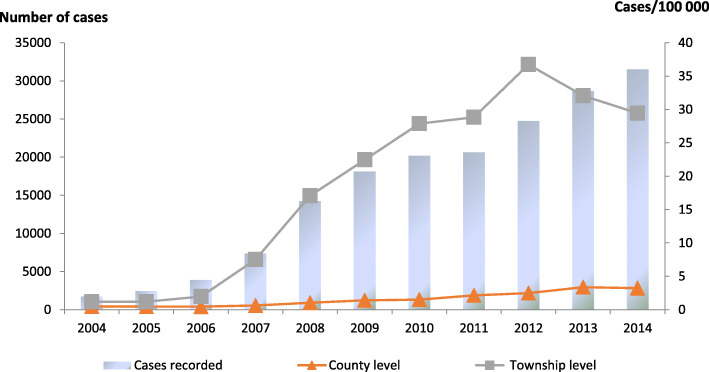
Distribution of patients by surgical interventions at different levels for 2004–2014

**Fig. 6:**
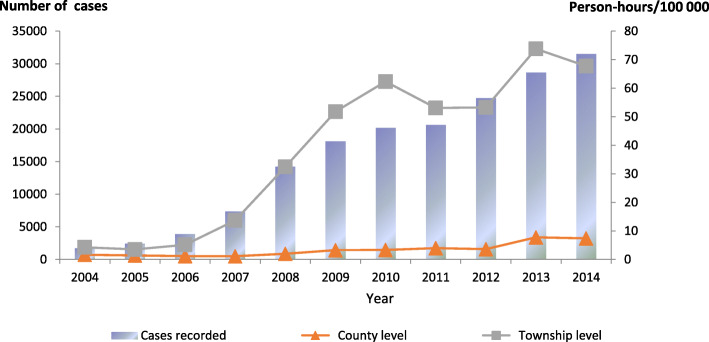
Patients treatment with albendazole at different levels for 2004–2014

Additionally, the number of surgical cases changed from 318 (18.2%) to 2379 (7.55%) from 2004 to 2014 (Table 1). A total of 121 patients (with co-infections) underwent surgical operations since 2010, and 513 unclassified cases were recorded since 2008. Moreover, 134 249 person-hours (the total time used for surgical treatment and drug treatment) were reported since 2010, including 74 145 person-hours for CE, 28833 for AE and 1802 for co-infection. However, 29 469 person-hours remained unclassified since 2008, particularly within the 2-year period 2011–2012 when the accumulated total person-hours were greater than 10 000. During these years, the mean prevalence for surgical patients after diagnosis as the patients at the county level (the project county) were 2.7 (CE), 0.1 (AE), 0.02 (co-infection) and 0.1 (unclassified) per 100 000 persons, respectively, while those at the township level were 2.9, 0.2, 0.04 and 0.13 per 100 000 persons, respectively. It should be noted that some patients were not suitable for surgery. The person-hours for the patients having received treatment were 10.2 (CE), 3.9 (AE), 0.3 (co-infection) and 4.4 (unclassified) at the county level, respectively, while the corresponding figures at the township level were 21.8, 8.5, 0.6 and 7.4, respectively (Table 2, Figs. 3, 4, 5 and 6).

**Table 2:** Patient interventions at different administrative levels in the areas endemic for echinococcosis during the period 2008–2014

Types	Mean prevalence (per 10^**5**^ population)							
	Patients subjected to surgery (Number of cases)				Case	Proportion (%)	Patients treated with albendazole (Number of person-hours)	
	Case	Proportion (%)	County level ***(95% CI***)	Township level (***95% CI***)			County level ***(95% CI***)	Township level (***95% CI***)
**CE**	10 053	88.4	2.71 (1.32–4.11)	2.94 (1.50–4.49)	74 145	55.2	*10.23 (4.85–15.61)*	*21.79 (9.44–31.43)*
**AE**	685	6.0	0.11 (0–0.24)	0.21 (0–0.46)	28 833	21.5	*3.93 (1.75–6.09)*	*8.52 (3.51–13.52)*
**Co-infection** ^**a**^	513	4.5	0.02 (0–0.06)	0.04 (0–0.09)	29 469	22.0	*0.25 (0–0.63)*	*0.56 (0–1.40)*
**Unclassified** ^**b**^	121	1.1	0.07 (0–0.15)	0.1 (0.01–0.3)	1802	1.3	*4.36(0–9.75)*	*7.41 (0–16.08)*

^a^ statistics from 2010 to 2014.^b^ statistics from 2008 to 2014. Person-hours: the total times after treatment for patients (surgical cases included)

*CE* cystic echinococcosis, *AE* alveolar echinococcosis

### Registered dogs intervention

Over the 10-year period from 2004 to 2014, the number of registered dogs tended to increase, i.e. reaching 28 times the previous annual number with a medium value of 115.0 (95% *CI*: 83.779–226.0), while the annual number of registered dogs already subjected to deworming increased 49 times with a medium value of 46.8 (95% *CI*: 35.9–151.1), respectively. In addition, the deworming coverage rate increased 73% in 2014 in comparison to 2005 with dogs being dewormed 6, 10 and 12 times per year based on an estimation according to the number of registered dogs during the period of 2012–2014 (Table 3). Because disease surveillance was not performed in the endemic regions due to the uneven development of disease control, the actual and accurate data of the dogs in which infection was not reported until 2012, with the exception of large-scale treatment schemes of the dog population using praziquantel tablets, whereas unpublished data based on *Echioncoccus* coproantigen ELISA tests (fecal antigen detection) for the average dog infection rates (available via the national pilot surveillance system) ranged between 2.89% (15 317/530 135) in 2013 and 3.35% (3944/117 671) in 2014 in the endemic areas.

**Table 3:** The situations for registered dogs deworming by the national echinococcosis control program from 2004 to 2014

Year	Registered dogs	Deworming cases	Coverage rate	Single deworming
	(×10 000)	(×10 000)	(%)	(×10 000)
2004	9.6	3.7	38.2	–
2005	23	2.4	10.2	–
2006	76.2	7.9	10.4	–
2007	93.2	38.9	41.5	–
2008	109.5	44.5	40.7	–
2009	115	46.8	40.7	–
2010	203.3	118.9	58.5	–
2011	216	176.9	81.9	–
2012	325.6	240.5^a^	73.9	1332.00
2013	263.1	170.3^a^	64.7	1714.20
2014	269	178.0^a^	66.2	2304.50

^a^ Based on statistical estimation. Coverage rate = deworming cases/registered dogs

--means no data

### Economics analysis

Over the 10-year period, a total costs of USD 110.7 million after discount was estimated for the national program by the National Ministry of Health, a sum that was verified by the health administration departments of the endemic areas. The direct total costs, ranging from albendazole treatment and surgical operations to deworming the dog population with oral praziquantel, were calculated at USD 12.3 million for humans and USD 15.8 million for dogs after discount by sum from 2004 to 2014 respectively. This means that in 2014, compared to 2006, the annual and accumulated intervention costs with respect to humans and dogs had increased 2840 times and 21.8 times, respectively. The annual cost proportion increased by 190% ([31.58–10.88]/10.88) and the accumulated costs proportion increased by 187% ([24.42–8.51]/8.51) in comparison to 2006, respectively (Table 4).

**Table 4:** Analysis of input-cost at the national level for patient and dog interventions from 2004 to 2014

Total input			Cost for patient and dog interventions				Proportion (%)	
Year	Annual	Accumulated	Patients	Dogs	Annual	Accumulated	Annual	Accumulated
2004	–	–	–	–	–	–	–	–
2005	30.1	30.1	–	–	–	–	–	–
2006	108.2	138.3	0.1	11.7	11.8	11.8	10.9	8.5
2007	392.3	530.6	51.0	55.6	106.6	118.3	27.2	22.3
2008	1038.4	1569.0	82.3	63.7	146.0	264.3	14.1	16.9
2009	1119.9	2688.9	112.5	67.6	180.01	444.9	16.1	16.5
2010	1709.3	4398.1	115.6	171.3	286.9	731.2	16.8	16.6
2011	1683.0	6081.1	168.2	254.3	422.6	1153.8	25.1	19.0
2012	1571.0	7652.1	168.7	346.2	515.0	1668.8	32.8	21.9
2013	1710.1	9362.2	252.0	243.6	495.6	2164.4	29.0	23.1
2014	1705.6	11 067.9	284.0	254.7	538.7	2703.1	31.6	24.4

State inputs and costs are expressed in USD 10 000. --means no data

### Estimated gaps

Although the current financial inputs did not fully adapt to the emerging number of patients and dogs in need of treatment and deworming, the gap narrowed by 68% in 2014, while it narrowed by 66% with respect to the accumulated costs in comparison to 2006, which is in accordance with the annual increases by China’s Central Government (Table 5).

**Table 5:** Input gap by estimation for patient and dog interventions during 2006–2014

Year	Input gap for patients		Input gap for dogs		Grand total proportion (%)	
	Annual	Accumulated	Annual	Accumulated	Annual	Accumulated
2006	0.3	0.3	100.8	100.8	90.0	90.0
2007	16.8	17.1	77.6	178.5	47.0	62.3
2008	17.0	34.1	92.9	271.4	43.0	53.6
2009	17.0	51.1	98.5	369.9	39.1	48.5
2010	9.3	60.4	121.6	491.5	31.3	43.0
2011	5.7	66.1	56.2	547.7	12.8	34.7
2012	20.0	86.1	122.4	670.1	21.7	31.3
2013	75.4	161.5	132.8	802.9	29.6	30.8
2014	89.4	250.9	130.1	933.0	29.0	30.5

Inputs are expressed in USD 10 000

## Discussion

Echinococcosis transmission mainly occurs in the pastoral and farming-pastoral regions of western China due to the complex animal populations and *Echinococcus* spp., which involve a wide range of intermediate and definitive hosts. Human epidemiological surveys may be carried out by serology, and ultrasonography is regarded as a simple, safe and reliable diagnostic tool for screening for internal cysts. Importantly, relatively safe and effective chemotherapy is available [20–29].

The data in this study demonstrate that the grand total person-hours spent on surgical interventions and treatment (including postoperative treatment) was 76.6% of the corresponding grand total proportion for the year of 2014, which was 1.2-fold compared to 2005. These results show a high disease burden in terms of DALYs in the endemic areas, which indicates that echinococcosis is a serious threat in parts of China. However, with the large-scale population screening, the patient numbers increased 18-fold in 2014 compared to 2004, while the prevalence per 100 000 population rate increased 17-fold at the county level and 10.8 times at the township level during the same time. Moreover, 1.5 times as many new cases were found, and the cumulative cases (expressed in person-hours) for surgical operations increased 4.2 times, which was 3.8 times the value for CE and 8.1 times the value for AE. For treatment (expressed in person-hours), the cumulative cases increased 5.7 times the value for the CE cases and 3.3 times the value for the AE cases for the last 5 years (2009–2014) compared to the former 5 years (2004–2008). In addition, increases of 0.6 times co-infection with surgical interventions and 0.5 times the co-infection cases with treatment for the last 3 years (2012–2014) were sustained compared to the former 2 years (2010–2011). For the unclassified cases, the cumulative cases were 13.7 times the value obtained with respect to surgical interventions and 6.3 times the value obtained for treatment for the last 4 years (2011–2014) compared to the former 3 years (2008–2010).

According to the mean prevalence rate at the county and township level, more diagnosed patients will be reported with a higher prevalence following a large-scale population screening; however, the characteristics of the co-infection cases and the unclassified cases treated during an additional 3-year period decreased by one-half. The results indicate that patients with co-infection and unclassified cases display an annual decreasing trend. Moreover, the patient treatments varied widely due to improvement of the diagnostic accuracy through ultrasound screening for the entire exposed population in high-risk areas. Therefore, patient diagnosis and management, including surgery and treatment with albendazole in endemic areas, are sustainable, whereas established surveillance systems for population prevalence and relevant factors need improvement at different levels, including compliance with respect to albendazole treatment, clinical follow-up, personal information and timely treatment updates of record.

Dogs are definitive hosts in the semi-domestic life cycle of *E. multilocularis* and play a key role due to their close association with *E. granulosus* throughout parasite transmission in the endemic areas of western China [11, 13, 30, 31]. Since 2005, the echinococcosis control initiative in China provides complete data for the management of registered dogs including deworming of infected dogs. Over the 7-year period of 2005–2011, the number of registered dogs increased 9-fold, but the deworming coverage rate only increased 8-fold in 2011 compared to the rate in 2005. However, according to the complete data records of registered dogs from 2012 to 2014, the coverage rate remained near 70% and exhibited an estimated 7-fold increase compared to 2005. These results were similar to those of the 2012–2014 period. Additionally, monthly anthelmintic treatment for dogs was more effective compared to previous studies [32, 33]. Data for the annual number of dogs deworming with dog-time calculated for large-scale deworming for the registered dogs increased 1.7-time in 2014 compared to 2012 and was estimated at 12 times per dog per year for 2014. In conclusion, the estimation of dog management and treatment over this period was performed at completely different levels on an annual timeline and helped reduce the parasite load to control the disease.

Notably, a strong relationship exists between patients and dogs, which means that there is a high risk that humans ingest parasite eggs directly through contact with infectious dogs or indirectly from contaminated environments. Studies also reveal that *E. granulosus* eggs remain viable and infective after 41 months that include warm summer and cold winter conditions [1, 2, 12, 13]. We also found that a positive correlation (*R* = 0.97, *P* < 0.01) between registered dogs with the available patients after Spearman’s correlation analysis of the present data. Currently, the management of stray dogs is particularly difficult in some regions where cultural acceptance is prevalent, several studies indicate that distribution of anthelmintic baits against wild and stray definitive hosts results in significant reductions in AE prevalence, particularly if innovative bait delivery is used [13, 34–37]. According to WHO reports regarding endemic regions, the human prevalence rates for CE can reach more than 50 per 100 000 person-years with prevalence levels as high as 5–10% worldwide; similar rates have been reported in regions of western China [1, 6, 17, 38, 39].

The national echinococcosis control program demonstrates that mass human population screening of echinococcosis, early detection and prompt treatment of human cases and large-scale dog deworming are valuable (particularly with respect to coverage) based on the cost proportion changes, with the exception of limitations of minor values based on the estimation. The results of the simple cost analysis show that over the 10-year period, a total cost of USD 27.0 million after discount on patients’ treatment and registered dogs deworming was accumulated. It reached 24.4% accounting for 1/4 of the total financial input, though currently they have not adapted to the emerging numbers of patients in need of treatment and dogs that should be dewormed. The annual and the accumulated costs increased 57-fold and 368-fold compared to those in 2005 respectively, and the grand total proportion narrowed by 70% with respect to annual or accumulated gaps, which is in accordance with the annual increase of the cost for patients and anti-parasite treatment of registered dogs.

In this study, due to the limitation of data confidentiality, we only collected data approved by the National Ministry of Health up to 2014, so the 4 years data after that were not covered and unable to include the distribution of wild canines (stray dogs and etc.) through the national program, which is a critical for blocking parasitic egg transmission. Moreover, although the national control programme was led by National Ministry of Health, the livestock management and vaccination were the responsibility of the Ministry of Agriculture and the outcomes will depend on their reports. In addition, the national surveillance system for echinococcosis was established in 2016, as there were hardly any historical data for analysis to fully reflect the prevalence for patients and dogs, except for the two national surveys in 2004 and 2012.

All in all, based on the results obtained through this study, we recommend that it will be more effectively on the national programme evaluation by multi-sectoral joints in the future, more attention should be paid to controlling wild canines during the ongoing program period and with increased focus on patients and dog interventions by the Chinese Government.

## Conclusions

This study showed that in endemic areas, patient diagnosis and management, dog management and treatment over this period helped reduce the parasite load to control the disease. Meanwile, it is suggested that more attention should be paid to controlling wild canines during the ongoing program period and sustainable follow-up evaluations by multi-sectoral joints are crucial for success and continued implementation of the national program.

## Data Availability

The supporting data in this paper are included in the context.

## Abbreviations

AE: Alveolar echinococcosis
CAAT: Cost for accumulated actual patients treatment
CAD: Cost for actual dogs deworming
CADD: Cost for accumulated actual dogs deworming
CAT: Cost for actual patient treatment
CE: Cystic echinococcosis
DALYs: Disability-adjusted life years
WHO: World Health Organization
